# 896. Epidemiology and effect of pandemic on regional coxsackie viral testing

**DOI:** 10.1093/ofid/ofad500.941

**Published:** 2023-11-27

**Authors:** Ramesh Kordi, Arthur Chang, Mark D Hicar

**Affiliations:** University at Buffalo, Buffalo, New York; University of Nebraska Medical Center, Omaha, Nebraska; University at Buffalo, Buffalo, New York

## Abstract

**Background:**

Coxsackie viruses (CV-A, CV-B), are a member of Picornaviridae of the genus Enterovirus and are commonly considered in summer and fall pediatric febrile illnesses. We noted an increase in CV testing post-pandemic and a lack of literature on the interpretation of seroprevalence and interpretation of CV testing.

**Methods:**

We accessed a regional health database (HEATHeLINK), between February 2017 and February 2023, and examined CV serum testing by Complement Fixation (CF). We analyzed data by age (21 years of age cutoff), month of testing, and compared testing pre-Covid-19 (February 2017-Feb 2020) and post-Covid-19 (March 2020-February 2023). We used the chi-square test to compare seropositivity between groups and SPSS version 28 for statistical analysis. CF titers ≥ 1:8 were considered positive.

**Results:**

901 serum CV-A tests were performed in 263 patients (median age 42, range 3-89) among whom 6 (2.3%) were positive for at least one serotype. No statistical difference was found between age groups (0.7% vs. 0.8%, p=0.9). The number of tests performed nearly doubled in the post-pandemic period with a notable lack of positive tests in the summer. 891 CV-B tests were performed in 238 patients (median age 41, range 3-80), among whom 129 (54.2%) were positive for at least one serotype. Positive test results were more notable in adults (26.7% vs. 37.7%, p=0.014). The testing had a higher rate of positive results pre-pandemic (176/381, 46.2% vs 145/510, 28.4%, p-value< 0.001). Tests and positive results were low for the first year of the pandemic (2020) compared to the following years, and the seasonality of testing and positive cases showed variability compared to pre-pandemic tests. In accessing a tertiary care center’s data, with a median age of 7 years old, testing was also notably increased post-pandemic (50 and 59 persons tested in 2021 and 2022 compared to an average of 13.5 cases pre-2021).

**Figure:**
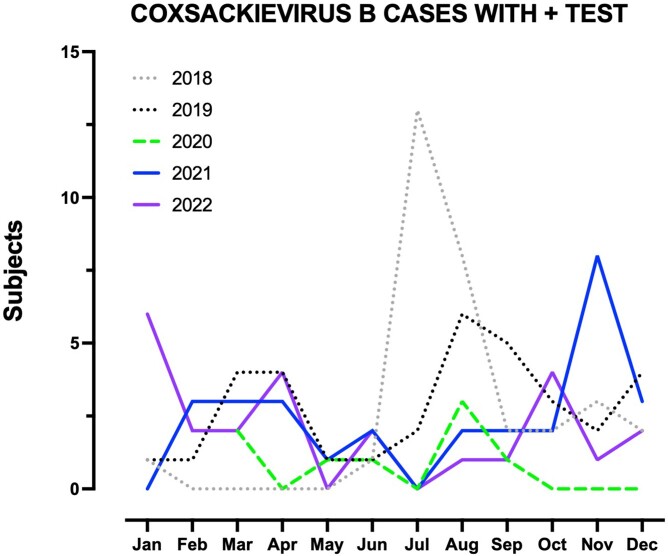

Data is from Western New York regional health database HEALTHeLINK. Gray and black dotted line represents two years preceding the pandemic and show normal seasonality pattern. The green dashed line shows that the number of positive cases during social distancing in our area, and blue and purple lines represent following years and display variability in cases clustering.

**Conclusion:**

Testing increased post-pandemic and results show variability in seasonality and utilization compared to pre-pandemic. Effects of social isolation and secondary viral circulation are potential explanations. Further detailed studies that include a higher level of confidence in diagnosis are warranted.

**Disclosures:**

**Mark D. Hicar, MD/PhD**, Pfizer: site investigator for 2 trial

